# Random forests for verbal autopsy analysis: multisite validation study using clinical diagnostic gold standards

**DOI:** 10.1186/1478-7954-9-29

**Published:** 2011-08-04

**Authors:** Abraham D Flaxman, Alireza Vahdatpour, Sean Green, Spencer L James, Christopher JL Murray

**Affiliations:** 1 Institute for Health Metrics and Evaluation, University of Washington, 2301 Fifth Ave., Suite 600, Seattle, WA 98121, USA; 2 Bill & Melinda Gates Foundation, Seattle, USA

**Keywords:** Verbal autopsy, cause of death certification, validation, machine learning, random forests

## Abstract

**Background:**

Computer-coded verbal autopsy (CCVA) is a promising alternative to the standard approach of physician-certified verbal autopsy (PCVA), because of its high speed, low cost, and reliability. This study introduces a new CCVA technique and validates its performance using defined clinical diagnostic criteria as a gold standard for a multisite sample of 12,542 verbal autopsies (VAs).

**Methods:**

The Random Forest (RF) Method from machine learning (ML) was adapted to predict cause of death by training random forests to distinguish between each pair of causes, and then combining the results through a novel ranking technique. We assessed quality of the new method at the individual level using chance-corrected concordance and at the population level using cause-specific mortality fraction (CSMF) accuracy as well as linear regression. We also compared the quality of RF to PCVA for all of these metrics. We performed this analysis separately for adult, child, and neonatal VAs. We also assessed the variation in performance with and without household recall of health care experience (HCE).

**Results:**

For all metrics, for all settings, RF was as good as or better than PCVA, with the exception of a nonsignificantly lower CSMF accuracy for neonates with HCE information. With HCE, the chance-corrected concordance of RF was 3.4 percentage points higher for adults, 3.2 percentage points higher for children, and 1.6 percentage points higher for neonates. The CSMF accuracy was 0.097 higher for adults, 0.097 higher for children, and 0.007 lower for neonates. Without HCE, the chance-corrected concordance of RF was 8.1 percentage points higher than PCVA for adults, 10.2 percentage points higher for children, and 5.9 percentage points higher for neonates. The CSMF accuracy was higher for RF by 0.102 for adults, 0.131 for children, and 0.025 for neonates.

**Conclusions:**

We found that our RF Method outperformed the PCVA method in terms of chance-corrected concordance and CSMF accuracy for adult and child VA with and without HCE and for neonatal VA without HCE. It is also preferable to PCVA in terms of time and cost. Therefore, we recommend it as the technique of choice for analyzing past and current verbal autopsies.

## Introduction

Verbal autopsy (VA) is a technique for measuring the cause-specific mortality burden for deaths that occur outside of hospitals. In VA, a trained interviewer collects detailed information on signs and symptoms of illness from laypeople familiar with the deceased. These interviews are analyzed by experts or by computer to estimate 1) the cause of death for each individual and 2) the distribution of causes of death in a population. This information can then be used by policy developers, donors, governments, or decision-makers to choose wisely in developing, requesting, and allocating health resources. For VA to provide useful information to individuals or to society, it is essential that the results of these interviews be mapped to the underlying cause of death accurately and quickly. Physician-certified verbal autopsy (PCVA) is currently the most common approach to mapping VA interviews to underlying cause of death, but this approach is expensive and time-consuming [1].

Machine learning (ML) methods are computer algorithms that infer patterns from examples [2]. In a classification task like VA analysis, an ML method processes a set of examples ("training data") that has gold standard classifications, and develops a model to classify additional data. Developing and refining ML methods is a vibrant area of research in computer science, and numerous new methods have been introduced over the past 50 years. One influential ML method, the artificial neural network (ANN), was applied to VA 10 years ago [3]. This approach was deemed potentially useful, pending further evaluation. By casting VA analysis as an application of general ML methods, incremental advances in ML techniques can be directly applied to improve the accuracy of VA analysis.

The Random Forest (RF) is an exciting innovation in ML technology [4]. The RF has been used extensively in many domains for classification tasks, and is consistently one of the top approaches [5]. Examples of using ML techniques in various domains include gene selection and classification of microarray data [6], modeling structural activity of pharmaceutical molecules [7], and protein interaction prediction [8]. For this study, we developed an application of the RF Method to VA analysis and compared the performance of RF to PCVA.

## Methods

### An overview of random forests

**Figure 1:**
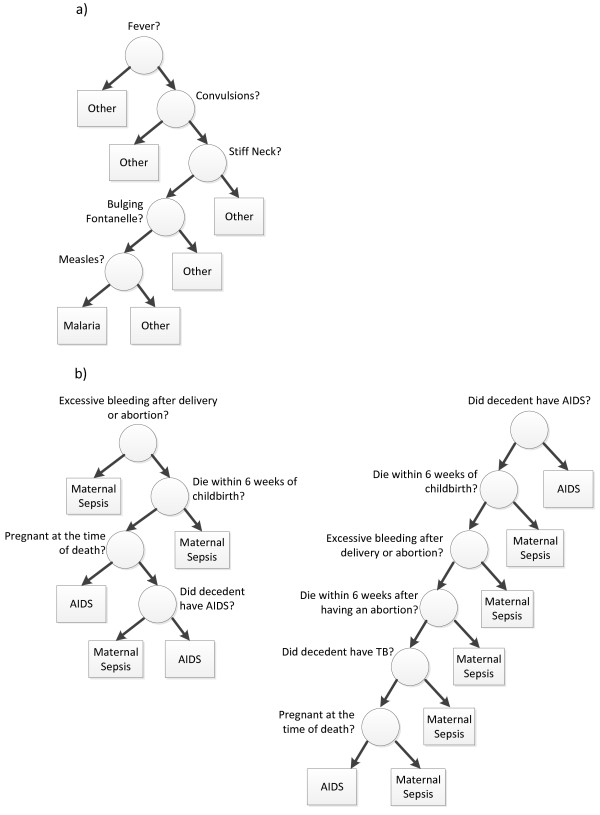
**Expert algorithm and RF decision trees**. A right branch from a node represents "yes" and a left branch represents "no." a) Decision tree representation of expert algorithm to identify malaria deaths in child VAs (one-versus-all approach); b) Two random decision trees generated by RF to distinguish AIDS deaths from maternal sepsis deaths (one-versus-one approach).

Our RF Method for VA analysis seems complicated at first, but is actually a combination of several simple ideas. The first of these is the "decision tree," a structure for representing a complex logical function concisely as branching decisions [9]. The decision trees in Breiman's Random Forest method are generated by a randomized algorithm from bootstrap-resampled training data, but the resulting trees are somewhat analogous to the expert algorithms used in early approaches to automatic VA analysis. In Figure 1, Panel a shows a decision-tree representation of an expert algorithm for deciding if a child death was due to malaria or other causes [10], while Panel b depicts decision trees generated as part of the random forest for distinguishing maternal sepsis from HIV deaths. In each, the decision between two possibilities is made by starting from the top level, and progressing to the next level following the branch to the right if the symptom at the current level was endorsed and to the left otherwise. For example, the expert algorithm in Figure 1a will only predict that the cause was malaria if the respondent said that the decedent had fever and convulsions and no stiff neck, no bulging fontanelle, and no measles.

Unlike expert algorithms, however, the decision trees in Breiman's Random Forest are generated automatically from labeled examples (the training dataset), without guidance from human experts. Instead, a random resampling of the training dataset is generated by drawing examples with replacement from the training dataset, and then a decision tree is constructed sequentially from this, starting from the root. At each node, the algorithm selects a random subset of signs and symptoms to consider branching on, and then branches on the one that best distinguishes between the labels for examples relevant to that node, halting when all relevant examples have the same label. Because of the randomness in this process, running the approach repeatedly on the same training dataset yields different trees, and two such trees are depicted in Figure 1b.

**Figure 2:**
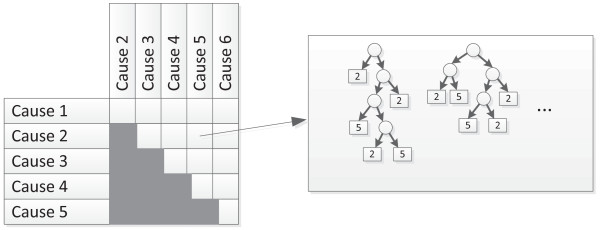
**Schematic representation of RF**.

Breiman's original formulation of RF proposed generating hundreds or thousands of decision trees this way, and then using them for prediction by calculating the prediction of each tree and taking a vote between their predictions. However, because of the long length of the cause list in verbal autopsy, we followed the "pairwise coupling" approach developed by Hastie [11]. We considered every pair of causes on the cause list, and generated 100 decision trees to distinguish between each pair. This resulted in a table of random forests, depicted schematically in Figure 2. The size of the forest was thus a function of the length of the cause list; for example, for the child VA module, the 21 causes produced a random forest of  trees.

**Figure 3:**
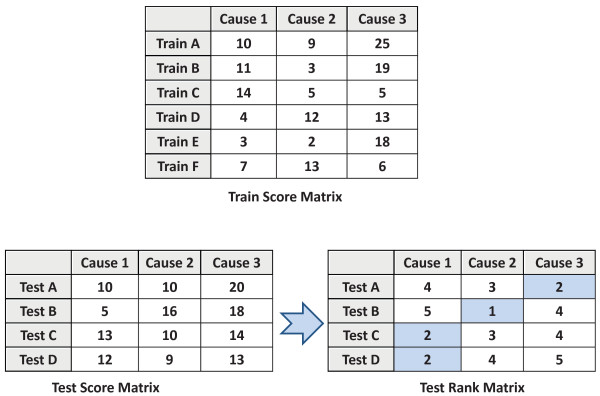
**Schematic representation of "ranking" technique for cause prediction from random forest scores**.

To aggregate the predictions of all of these trees, we tallied cause-specific scores by counting the number of trees that predicted each cause. We then normalized the score for each cause using a novel ranking procedure. The complete process of mapping from scores through ranks to predictions is demonstrated in Figure 3, where, for example, Test C is predicted to be caused by Cause 1, which is not the highest scored cause for this example, but is the highest *ranked *cause. The full process is as follows: the Test Score Matrix is converted to a Test Rank Matrix on an entry-by-entry basis, by finding the rank of each entry among the corresponding column in the Train Score Matrix. For example, Test A, Cause 3 has score 20, which is the second-highest score when compared with the Cause 3 column of the Train Score Matrix, so it has a rank of 2 in the Test Rank Matrix. After Test A had Cause 1 and Cause 2 ranked similarly, the procedure predicted that Test A was caused by Cause 3 because this is the cause that was highest ranked for A. This is a nonparametric form of whitening, which makes the scores for different causes directly comparable. This approach has a natural generalization to predicting multiple causes for a single death, where the second-highest ranked cause is predicted as the second most likely, etc.

### Validation using the PHMRC gold standard test/train datasets

The Population Health Metrics Research Consortium (PHMRC) gold standard verbal autopsy validation study provides a large multisite dataset to assess the performance of new or existing verbal autopsy methods. The PHMRC study identified deaths that met defined clinical diagnostic criteria for cause of death. Then, interviewers visited the households of the deceased to conduct full verbal autopsies. Thus, the gold standard cause of death is paired with the responses from a verbal autopsy. The numbers of records from each site are provided in Table 1. As part of the PHMRC study, all variables including free-text were converted into a series of dichotomous items. All aspects of the study are described elsewhere in more detail [12]. Additional files 1, 2, and 3 list the 40 most informative variables for each cause in the adult, child, and neonatal modules after this data preparation phase was completed.

**Table 1:** Numbers of VAs collected by site and gold standard level

Site	Adult		Child		Neonate		Total
	Level 1	Level 2	Level 1	Level 2	Level 1	Level 2	
Andhra Pradesh, India	1285	269	385	66	376	1	**2,382**
Bohol, Philippines	998	262	234	30	374	0	**1,898**
Dar es Salaam, Tanzania	1556	162	366	106	1047	2	**3,239**
Mexico City, Mexico	1373	215	124	4	313	2	**2,031**
Pemba Island, Tanzania	266	31	156	105	261	3	**822**
Uttar Pradesh, India	1277	142	412	87	251	1	**2,170**
**Total**	**6,755**	**1,081**	**1,677**	**398**	**2,622**	**9**	**12,542**

Murray et al. have shown that many traditional metrics of performance, such as specificity or relative and absolute error in CSMFs, are sensitive to the CSMF composition of the test dataset [13] and recommend that robust assessment of performance be undertaken on a range of test datasets with widely varying CSMF compositions. Further, metrics of individual concordance need to be corrected for chance to adequately capture how well a method does over random or equal assignment across causes.

The PHMRC has developed a set of 500 test/train splits of the data, which we analyzed. The splits were generated randomly, stratified by cause. Each has a random 75% of examples of each cause in the training set and 25% in the test set. For each split, we used the training data to generate random forests for each pair of causes and then we applied these forests to the test dataset. We never allowed contamination between the training data and the test data - they were kept strictly separate in all steps of the analysis. Further, the cause composition of the test dataset is based on a random draw from an uninformative Dirichlet distribution. The Dirichlet distribution specifies random fractions that sum to 1. Each test split is resampled with replacement to meet the cause fractions specified by a Dirichlet draw. Consequently, each test split has a different distribution of cause fractions, and the cause composition of the training data and test data are always different.

We assessed the performance of RF at assigning individual causes of death using median chance-corrected concordance by cause across the 500 test datasets and the median average chance-corrected concordance across causes in the 500 test datasets, following the recommendations of Murray et al [13]. For assessing the performance of RF in estimating CSMFs, we calculated the median CSMF accuracy as well as slope, intercept, and root mean squared error (RMSE) of a linear regression for each cause as a summary of the relationship between estimated CSMFs for a cause and the true CSMF in a particular test dataset [13]. We benchmark RF against PCVA on the same dataset using the results reported by Lozano et al [14].

Murray et al. analyzed data in China two ways: including all items and excluding items that reflected the decedent's health care experience (HCE) [15]. The purpose of excluding the HCE items is to assess how RF would perform on VA for communities without access to health care. They found, for example, that a considerable component of PCVA performance was related to the household recall of hospital experience or availability of a death certificate or other records from the hospital. We assessed the performance of RF in adults, children, and neonates both with and without the free-response items and the structured questions that require contact with health care to answer (marked in Additional files 1, 2, and 3).

There are many potential variations in implementing RF. Specifically:

• Continuous and categorical variables can be included as is, or can be dichotomized to reduce noise

• The training data can be reweighted so that all causes are represented equally or left as is

• Decision trees can compare cause *j *to all other causes at once, or compare cause *j *to each other individual cause to come up with "votes"

• The signal-to-noise ratio can be improved by removing low-information items using the Tariff Method [16], or all items can be used

• Different numbers of signs and symptoms can be used at each decision node

• Different numbers of trees can be used in the forest

• Cause assignment can be based on the highest scoring cause for each death or on ranking the scores and assigning to the cause with the highest rank

We conducted an extensive sensitivity analysis to understand the importance of decisions between levels of Tariff-based item reduction, the choice of number of signs and symptoms at every decision node (*m*), the choice of number of trees (*n*) in each one-versus-one cause classification, and the difference between max-score and max-rank cause assignment. To avoid overfitting the data when selecting between the model variants, we conducted our sensitivity analysis using splits 1 to 100 and repeated the analysis using splits 101 to 200 and a random subset of 50 splits. The results of the sensitivity analysis are included in Additional file 4 and show that cause assignment by rank is superior to assignment by score but that the other parameters do not affect chance-corrected concordance or CSMF accuracy. The results shown in the next section are all for the one-versus-one model, with dichotomized variables, with training data reweighted to have equal class sizes, using the 40 most important Tariff-based symptoms per cause, *m *= 5, *n *= 100, and the max-rank cause assignment, which produced the highest CSMF accuracy for seven of the first 200 splits of the child VA data with HCE and the highest chance-corrected concordance for 14.

## Results

### Individual cause assignment compared to PCVA

Table 2 shows that, for RF over 500 splits, the median value of average chance-corrected concordance for adult VAs without HCE was 37.7% (95% uncertainty interval [UI]: 37.6%, 38%), and for adult VAs with HCE it was 48% (47.8%, 48.2%); for child VAs without HCE it was 46.5% (46.1%, 47%), and for child VAs with HCE it was 51.1% (50.7%, 51.6%). For neonatal VAs without HCE the median average chance-corrected concordance was 33.5% (33%, 33.9%), and for neonatal VAs with HCE it was 34.9% (34.5%, 35.4%). Note that the neonate VAs results presented in the tables for PCVA are for a shorter cause list that only includes six causes, where all the preterm delivery causes are grouped together. This is due to the fact that PCVA performed very poorly on a cause list with 11 causes.

**Table 2:** Median chance-corrected concordance (%) for RF and PCVA, by age group with and without HCE

		RF		PCVA	
		Median	95% UI	Median	95% UI
**Adult**	**No HCE**	37.7	(37.6, 38.0)	29.7	(29.4, 29.8)
	**HCE**	48.0	(47.8, 48.2)	44.6	(44.3, 44.8)
**Child**	**No HCE**	46.5	(46.1, 47.0)	36.3	(35.9, 36.6)
	**HCE**	51.1	(50.7, 51.6)	47.8	(47.1, 48.3)
**Neonate**	**No HCE**	33.5	(33.0, 33.9)	27.6	(27.2, 28.0)
	**HCE**	34.9	(34.5, 35.4)	33.3	(32.8, 33.7)

The differential value of HCE to RF in adult VA is more substantial than in child or neonatal VAs. Including HCE responses yields a significant relative increase of 10.3% in median chance-corrected concordance for adult VA. This could be because adults have more substantial experience with health care, and hence more relevant information is generated that aids in VA analysis, or it could be confounded by the differences between the adult, child, and neonate cause lists. In PCVA, however, including HCE responses produces a large increase in median chance-corrected concordance for all modules. In all six of these settings, the median chance-corrected concordance is significantly higher for RF than for PCVA.

**Figure 4:**
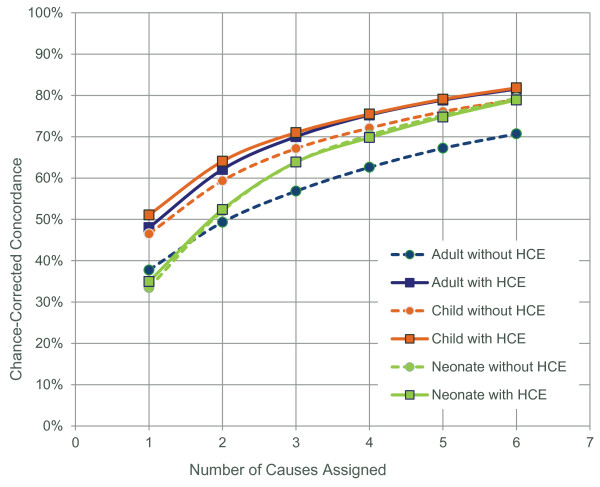
**Partial-cause assignment increases partial chance-corrected concordance for adult, child, and neonate VAs with and without HCE**. Slope of increase is higher between one and two cause assignments.

Figure 4 shows that partial-cause assignment increases the partial-cause chance-corrected concordance for all age groups with and without HCE. The increasing partial-cause chance-corrected concordance as a function of the number of causes shows that RF contains additional information in the second, third, etc., most likely causes. However, as the partial-cause assignment continues, the added value from new cause assignment decreases due to the chance-correcting element in the partial-chance-corrected concordance formula, as demonstrated by the decreasing slope.

**Figure 5:**
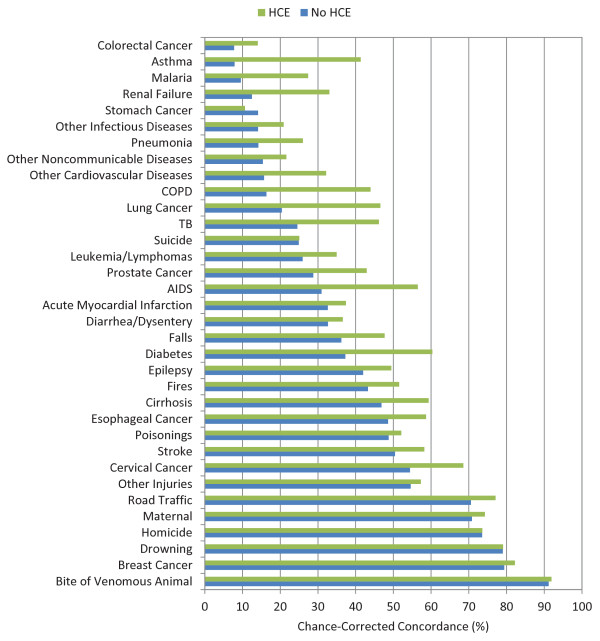
**Median chance-corrected concordance (%) for RF across 500 splits, by cause, for adult VA, with and without HCE**.

**Figure 6:**
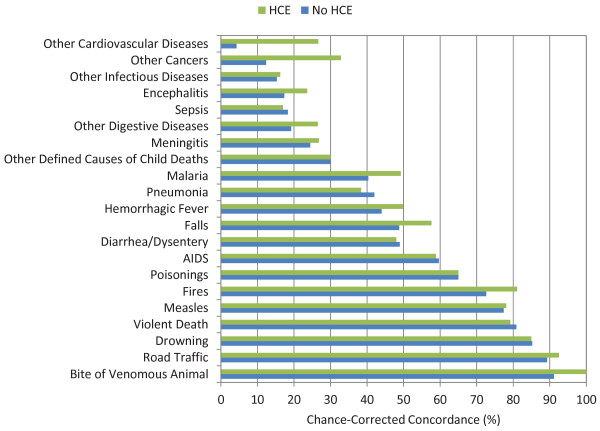
**Median chance-corrected concordance (%) for RF across 500 splits, by cause, for child VA, with and without HCE**.

**Figure 7:**
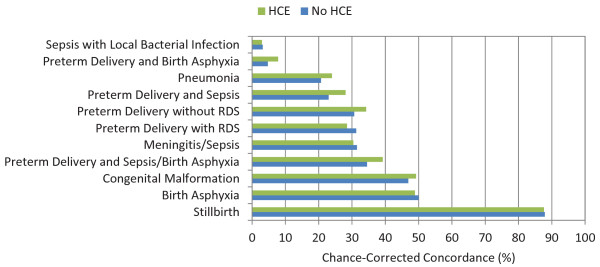
**Median chance-corrected concordance (%) for RF across 500 splits, by cause, for neonatal VA, with and without HCE**.

**Figure 8:**
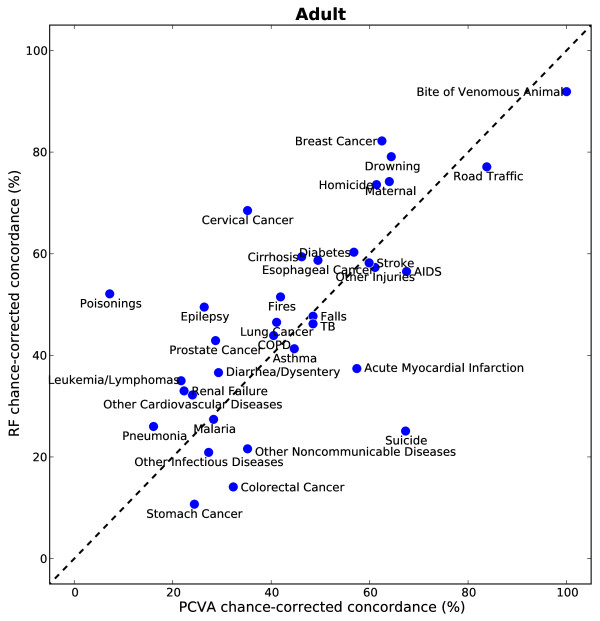
**Scatter of median chance-corrected concordance of RF versus PCVA, for adult module**.

**Figure 9:**
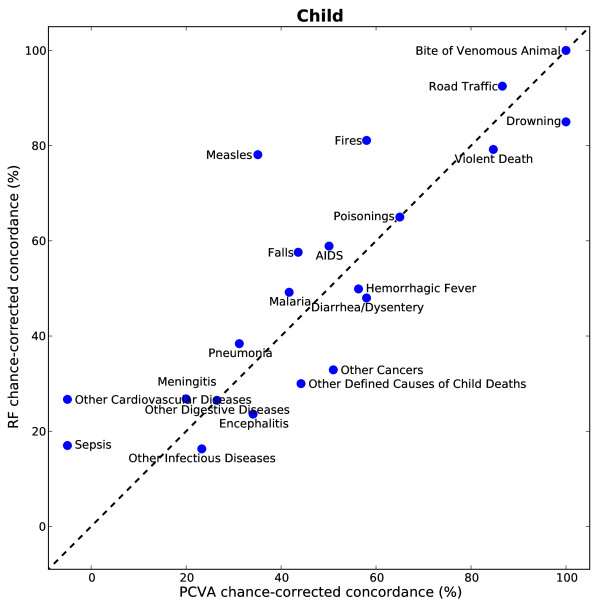
**Scatter of median chance-corrected concordance of RF versus PCVA, for child module**.

Figures 5, 6, and 7 show the chance-corrected concordance of RF on a cause-by-cause basis for adult, child, and neonatal VAs with and without HCE (also see Additional file 5). Figure 8 shows that on a cause-by-cause basis, RF is better than PCVA with HCE by at least 10 percentage points of chance-corrected concordance for 13 causes for adult deaths (lung cancer, fires, renal failure, pneumonia, homicide, drowning, cirrhosis leukemia/lymphomas, breast cancer, prostate cancer, epilepsy, cervical cancer, and poisonings). On the other hand, PCVA performed substantially better in detecting suicide, acute myocardial infarction, stomach cancer, other noncommunicable diseases, and AIDS. In addition, as depicted in Figure 9, in five causes of child deaths, RF concordance is at least 10 percentage points higher with HCE (falls, sepsis, fires, other cardiovascular diseases, and measles). Among causes of child deaths, PCVA performed better in detecting other cancers, drowning, encephalitis, violent death, diarrhea/dysentery, and other defined causes of child deaths. Head-to-head comparison of the neonatal performance between PCVA and RF is not possible though, as PCVA utilized a shorter cause list.

Another advantage of RF over PCVA is its relatively consistent performance in the presence and absence of HCE variables. PCVA concordances vary significantly with absence of HCE variables (e.g., for 22 causes of adult deaths, without HCE, concordance decreased by more than 10 percentage points). On the other hand, RF concordance only decreases substantially in 15 adult causes. In addition, RF shows more consistency among all causes. For example, its minimum median chance-corrected concordance in adult causes is 7.9% (without HCE) and 10.7% (with HCE), while minimum median chance-corrected concordance for PCVA without HCE is negative for two causes (meaning PCVA did worse than chance). RF does benefit substantially from HCE variables for certain important causes, however. For example, for adult deaths due to tuberculosis, AIDS, diabetes, and asthma, chance-corrected concordance increased by more than 20 percentage points when HCE variables were included.

### CSMF estimation compared to PCVA

Table 3 compares the median CSMF accuracy for RF and PCVA. Over 500 splits, the median value of CSMF accuracy for RF for adult VAs with HCE was 0.772 (0.769, 0.776), and for adult VAs without HCE it was 0.726 (0.721, 0.730); for child VAs with HCE it was 0.779 (0.775, 0.785), and for child VAs without HCE it was 0.763 (0.755, 0.769); for neonatal VAs with HCE it was 0.726 (0.717, 0.734), and for neonatal VAs without HCE it was 0.720 (0.71, 0.732). The patterns for this population-level estimation quality metric are qualitatively the same as those observed in the individual-level metric above. The value of HCE information is more substantial for adult VA, although it yielded a smaller increase, changing the median CSMF accuracy by 0.046. For child VA, the value is small, where it yields an increase of 0.016, and for neonate, the HCE value is not significant (increase of 0.006). In all of these settings except for neonates with HCE, the median CSMF accuracy was significantly higher for RF than for PCVA. For the neonates with HCE, the difference was not statistically significant, and the comparison was done for a six cause list for PCVA and a more challenging 11 cause list for RF.

**Table 3:** Median CSMF accuracy for RF and PCVA, by age group with and without HCE

		RF		PCVA	
		Median	95% UI	Median	95% UI
**Adult**	**No HCE**	0.726	(0.721, 0.730)	0.624	(0.619, 0.631)
	**HCE**	0.772	(0.769, 0.776)	0.675	(0.669, 0.680)
**Child**	**No HCE**	0.763	(0.755, 0.769)	0.632	(0.626, 0.642)
	**HCE**	0.779	(0.775, 0.785)	0.682	(0.671, 0.690)
**Neonate**	**No HCE**	0.720	(0.710, 0.732)	0.695	(0.682, 0.705)
	**HCE**	0.726	(0.717, 0.734)	0.733	(0.719, 0.743)

**Figure 10:**
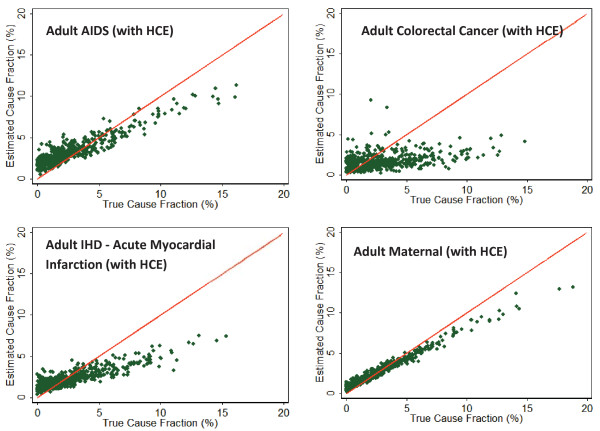
**Estimated versus true CSMFs for 500 Dirichlet splits, showing that for selected causes of adult mortality (AIDS, colorectal cancer, maternal, and IHD), the performance of RF varies**. For AIDS and IHD, RF tends to overestimate the cause fraction when the true CSMF is small and underestimate otherwise. For colorectal cancer, RF mostly assigns the same CSMF regardless of true CSMF, and for maternal causes, RF is more accurate.

Figure 10 shows scatter plots of the estimated versus true CSMF for four select causes of adult deaths (each of the 500 splits contributes a single point to the scatter). The figure shows how RF estimation quality tends to be different for different causes. As depicted, RF estimations for AIDS, maternal, and ischemic heart disease (IHD) are closely correlated with the true CSMFs. However, for colorectal cancer, estimations are noisier, and regardless of the true CSMF, RF assigns similar CSMFs in all 500 splits. To summarize the quality of RF estimation for each cause for all age groups, Additional file 6 shows the slope, intercept, and RMSE from linear regression of estimated versus true CSMFs. This population-level metric of analysis quality gave results qualitatively similar to the individual-level metric on a cause-specific basis. The RF CSMF slopes range from 0.097 to 0.904 for adult VAs, 0.105 to 0.912 for child VAs, and 0.079 to 0.845 for neonatal VAs. PCVA has similar ranges for the three age groups. However, on a cause-by-cause basis, PCVA and RF show different characteristics. A comparison revealed that, for the same causes that the methods have high chance-corrected concordance, the CSMF regression slope is higher for RF. This shows that RF attains higher cause-specific chance-corrected concordances as a result of better classification, not simply by assigning a higher portion of deaths to some causes.

The results of performing RF with a higher number of trees in each one-versus-one cause classifier showed that the method is stable by only using 100 trees per classifier. It should be noted that, while in the literature it is suggested that increasing the number of trees increases the classification precision, as our overall RF Method includes an ensemble of one-versus-one classifiers (e.g., for adult VAs, RF has  one-versus-one classifiers, each including 100 trees), the overall number of trees is high, which results in stable performance.

## Discussion

We found that the RF Method outperforms PCVA for all metrics and settings, with the exception of having slightly lower CSMF accuracy in neonates when HCE was available. Even in this single scenario, the difference in CSMF accuracy is not statistically significant, and furthermore, the PCVA analysis for neonates was limited to a six cause list, while the RF analysis was done on the full 11 cause list. The degree of the improvement varies among metrics, among age modules, and with the presence or absence of HCE variables. When the analysis is conducted without HCE variables, RF is particularly dominant.

The superior performance of RF compared to PCVA with respect to all of our quality metrics is excellent because this method also reduces cost, speeds up the analysis process, and increases reliability. While it may take days for a team of physicians to complete a VA survey analysis, a computer approach requires only seconds of processing on hardware that is currently affordably available. In addition, using machine learning leads to reliability, since the same interview responses will lead to the same cause assignment every time. This is an important advantage over PCVA, which can produce results of widely varying quality among different physicians, according to their training and experience [14].

Despite these strengths of RF, the method does have weaknesses in individual-level prediction of certain causes. For example, chance-corrected concordances for malaria and pneumonia in adults are around 25% even with HCE. Chance-corrected concordances for encephalitis, sepsis, and meningitis in children are in the 15% to 25% range. However, in many applications, it is the population-level estimates that are most important, and the linear regression of true versus estimated cause fraction shows that for these causes, RF has a RMSE of at most 0.009 for the adult causes and 0.02 for the child causes. It may be possible to use these RMSEs together with the slopes and intercepts to yield an adjusted CSMF with uncertainty.

While the ANN method used by Boulle et al. 10 years ago [3] showed the potential of using ML techniques, the RF Method we have validated here has proven that ML is ready to be put into practice as a VA analysis method. ML is an actively developing subdiscipline of computer science, so we expect that future advances in ML classification will be invented over the coming years, and VA analysis techniques will continue to benefit from this innovation. During the development of our approach, we considered many variants of RF. However, the possibilities are endless, and even some other variant of RF may improve on the method presented here. For example, nonuniformly increasing the number of trees in the forest to have proportionately more for select causes (in the spirit of Boosting [17]) is a potential direction for future exploration.

For any ML classifier to be successful, several requirements should be met. As discussed earlier, the accuracy of classification relies considerably on the quality of the training data (deaths with gold standard cause known to meet clinical diagnostic criteria). While the PHMRC study design collected VA interviews distributed among a wide array of causes from a variety of settings, certain causes were so rare that too few cases occurred to train any ML classifier to recognize them. Future studies could focus on collecting additional gold standard VAs for priority diseases to complement the PHMRC dataset. These additional data could improve the accuracy of RF and other ML models on certain selected causes. Future research should also focus on assessing VA's performance in different settings. For example, users in India may be interested specifically in how RF performs in India instead of across all of the PHRMC sites, particularly if it is possible to train the model only on validation deaths from India.

All VA validation studies depend critically on the quality of validation data, and this RF validation is no exception. A unique feature of the PHMRC validation dataset, the clinical diagnostic criteria, ensures that the validation data are very precise about the underlying cause of death. However, this clinical diagnosis also requires that the deceased have some contact with the health system. The validity of the method therefore depends critically on the assumption that the signs and symptoms observed in the deaths that occur in hospitals for a given cause are not substantially different than deaths from that cause that occur in communities without access to hospitals. We have investigated this assumption by conducting our analysis with and without HCE items, which gives some indication of the potential differences.

The machine learning technique described in this paper will be released as free open source software, both as stand-alone software to run on a PC and also as an application for Android phones and tablets, integrated into an electronic version of the VA instrument.

## Conclusions

We presented an ML technique for assigning cause of death in VA studies. The optimization steps taken to improve the accuracy of RF classifiers in VA application were presented. We found that our RF Method outperformed PCVA in chance-corrected concordance and CSMF accuracy for adult and child VA with and without HCE and for neonatal VA without HCE. In addition, it is preferable to PCVA in terms of both cost and time. Therefore, we recommend it as the technique of choice for analyzing past and current verbal autopsies.

## Abbreviations

ANN: artificial neural network; CCVA: computer-coded verbal autopsy; CSMF: cause-specific mortality fraction; VA: verbal autopsy; ML: machine learning; PCVA: physician-certified verbal autopsy; PHRMC: Population Health Metrics Research Consortium; RF: Random Forest; RMSE: root mean squared error; HCE: health care experience; IHD: ischemic heart disease.

## Competing interests

The authors declare that they have no competing interests.

## Authors' contributions

ADF, AV, and SG performed analyses. AV and CJLM edited the manuscript. SLJ helped prepare the data and results. ADF drafted the manuscript and approved the final version. ADF accepts full responsibility for the work and the conduct of the study, had access to the data, and controlled the decision to publish. All authors have read and approved the final manuscript.

## Supplementary Material

Additional file 1 Top 40 items based on Tariff Method for adult causes (Items that are extracted from open text field are marked with *, other HCE variables are marked with +). Click here for file

Additional file 2 Top 40 items based on Tariff Method for child causes (Items that are extracted from open text field are marked with *, other HCE variables are marked with +). Click here for file

Additional file 3 Top 40 items based on Tariff Method for neonate causes (Items that are extracted from open text field are marked with *, other HCE variables are marked with +). Click here for file

Additional file 4 **Sensitivity analysis for 54 variants of the RF algorithm applied to 200 splits of the child VA data with HCE**. Click here for file

Additional file 5 **Median chance-corrected concordance (%) across 500 splits, by age group and cause with and without HCE**. Click here for file

Additional file 6 **Slope, intercept, and RMSE for linear regression of true versus estimated CSMF**. Click here for file
